# Current practices and perceived implementation barriers for working with alcohol prevention in occupational health services: the WIRUS OHS study

**DOI:** 10.1186/s13011-019-0217-2

**Published:** 2019-06-26

**Authors:** Mikkel Magnus Thørrisen, Jens Christoffer Skogen, Ingvild Kjeken, Irene Jensen, Randi Wågø Aas

**Affiliations:** 1 Department of Occupational Therapy, Prosthetics and Orthotics, Faculty of Health Sciences, OsloMet – Oslo Metropolitan University, PO box 4 St. Olavs plass, NO-0130 Oslo, Norway; 2 Presenter – Making Sense of Science, PO box 8118, NO-4068 Stavanger, Norway; 3 Department of Health Promotion, Norwegian Institute of Public Health, PO box 973, NO-5808 Bergen, Norway; 4 Center for Alcohol & Drug Research, Stavanger University Hospital, PO box 8100, NO-4068 Stavanger, Norway; 5 Department of Public Health, Faculty of Health Sciences, University of Stavanger, PO box 8600, Forus, NO-4036 Stavanger, Norway; 6 National Advisory Unit on Rehabilitation in Rheumatology, Diakonhjemmet Hospital, PO box 23, Vindern, NO-0319 Oslo, Norway; 7 Institute of Environmental Medicine, Division of Intervention and Implementation Research for Worker Health, Karolinska Institutet, SE-171 77 Stockholm, Sweden

**Keywords:** Alcohol consumption, Occupational health services, Workplace interventions, Workforce, Implementation, Prevention

## Abstract

**Background:**

Alcohol is associated with detrimental health and work performance outcomes, and one to three out of ten employees may benefit from interventions. The role of occupational health services (OHS) in alcohol prevention has received little attention in research. The primary aims of this study were to explore current practices of alcohol prevention targeting employees in occupational health settings, and examine whether and which perceived implementation barriers were associated with alcohol prevention activity. The secondary aim was to explore whether barriers were differentially associated with primary, secondary and tertiary prevention activities.

**Methods:**

In this cross-sectional study, survey data were collected from 295 OHS professionals in Norway in 2018. Data were analysed by means of descriptive statistics, one-way analysis of variance, paired samples t-tests, and multivariate linear regression analyses.

**Results:**

Overall, seven out of ten OHS professionals worked with alcohol-related cases less than monthly, while only one out of ten did so on a weekly basis. Their activities were more focused on tertiary prevention than on primary and secondary prevention. Physicians, psychologists and nurses reported to handle alcohol-related issues more often than occupational therapists and physical therapists. Higher levels of implementation barriers internal to the OHS’ organisation (competence, time and resources) were associated with lower alcohol prevention activity. Barriers external to the OHS’ organisation (barriers concerning employers and employees) were not. This pattern was evident for primary, secondary and tertiary prevention activities. A majority of OHS professionals agreed that employees’ alcohol consumption constitute a public health challenge, and that OHS’ should focus more on alcohol prevention targeting employees.

**Conclusions:**

Occupational health settings at workplaces may be particularly serviceable for alcohol prevention programmes since the majority of the population is employed and the majority of employees consume alcohol. An increase in overall prevention activity, and a shift from mainly focusing on tertiary prevention to an increased emphasis on primary and secondary prevention, may both hinge on increased training of OHS professionals, emphasising knowledge on the importance of working with alcohol prevention, and training in administering alcohol prevention programmes. Making alcohol prevention a priority may also require increased allocation of time and resources.

**Electronic supplementary material:**

The online version of this article (10.1186/s13011-019-0217-2) contains supplementary material, which is available to authorized users.

## Background

Occupational health services (OHS) aim to protect and promote employees’ safety and health, as well as to improve the work environment and working conditions [1–3]. The majority of the population is employed and the majority of employees consume alcohol. Therefore, several researchers have argued that the OHS should be more actively involved in alcohol prevention targeting employees [1, 4–6]. It has proved feasible to conduct brief alcohol prevention programmes as an integrated part of regular health examinations routinely performed within the OHS [7, 8], and early identification and interventions targeting problem drinking may even be considered more appropriate in OHS as compared to specialised health care [9]. In a Swedish study [1], it was discovered that OHS professionals were generally interested in gaining further training and knowledge regarding alcohol prevention.

Harmful alcohol consumption is a major risk factor for disease, disability and mortality, and has been identified as a causal agent in more than 200 disease and injury conditions [10, 11]. According to the World Health Organization (WHO) [12], harmful alcohol consumption is related to approximately three million annual deaths globally. A recent study from the Global Burden of Disease project [13], based on data from 694 individual/population-level sources and 592 prospective and retrospective studies, found that alcohol consumption is the leading risk factor for deaths and disability-adjusted life-years among the population aged 15 to 49 years (accounting for 3.8% of female deaths and 12.2% of male deaths). Despite robust evidence for adverse health consequences attributable to alcohol consumption, some studies have found a J-shaped relationship between alcohol and health, indicating that low to moderate consumption levels may carry certain health benefits. Moderate consumption has been inversely related to risk for certain cardiovascular diseases [14], diabetes type 2 [15] and certain mental health outcomes [16]. Such findings suggest that potential health benefits should be weighted against risks [17]. It is, however, somewhat unclear whether such results reflect true protective effects of alcohol or is a result of confounding [18, 19]. Nevertheless, decades of evidence implies that potential health benefits from alcohol will be outweighed by adverse consequences [11–13, 20]. Hence, efforts to reduce overall population-level alcohol consumption should be emphasised [13].

**Fig. 1:**
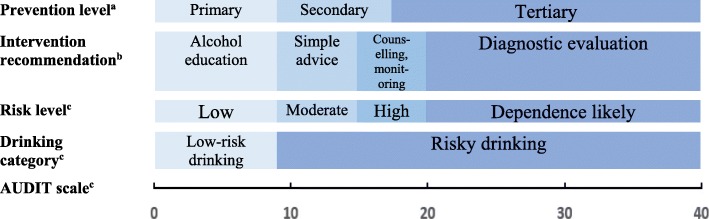
Conceptual model of the relationships between alcohol consumption, drinking categories, risk levels, intervention recommendations and prevention levels. ^a^Based on [32]; ^b^Based on [31, 33]; ^c^Based on [31]

Alcohol is by far the most used psychoactive substance in the workforce [21]. One may discriminate between workforce alcohol consumption (overall consumption, regardless of context; [21]) and work-related alcohol consumption (consumption during working hours, shortly prior to work, or in contexts related to the work environment; [21–24]). Three out of four employees have been found to be overall regular drinkers, while approximately one out of ten has consumed alcohol during working hours [21]. In a Norwegian study, it was found that 43% of regular drinkers’ consumption occurred in work-related settings [25]. Studies have estimated that one to three out of ten employees may benefit from alcohol prevention programmes [25–30]. Both in research and in policy guidelines, attempts have been made to distinguish between low-risk and risky drinking. Risky drinking has been defined as a pattern of drinking that increases the risk of social, legal, medical, occupational, domestic and economic problems [31]. Figure 1 presents a conceptual model for the relationships between alcohol consumption, drinking categories, prevention levels, risk levels and intervention recommendations.

Based on WHOs Alcohol Use Disorders Identification Test (AUDIT), an individual’s drinking pattern may be measured on a scale ranging from 0 to 40 [31, 34]. A sum score of eight or higher is generally considered the threshold for risky drinking [31, 35]. Moreover, risky drinking is categorised into three risk levels (moderate risk: scores 8–15; high risk: scores 16–19; and dependence likely risk: scores 20–40) [31]. According to WHOs international intervention guidelines [33], low-risk drinkers should receive information about alcohol use and potential negative consequences, moderate-risk drinkers may benefit from low-cost interventions such as simple advice, high-risk drinkers should receive brief counselling and consecutive monitoring, while those with likely alcohol dependence should be referred to further diagnostic evaluation. In accordance with Coohey and Marsh’s [32] conceptualisations of prevention levels, low-risk drinking employees constitute the target group for primary prevention activities, i.e., activities aimed at preventing an undesirable end-state (alcohol-related problems) before it occurs (or aimed at maintaining low-risk drinking as a desirable state). Secondary prevention activities target individuals experiencing the early phases of the undesirable end-state (employees with moderate to high risk), while tertiary prevention is focused on employees with high to dependence likely risk [32].

Employees’ alcohol consumption carries substantial societal costs. Productivity impairments associated with alcohol consumption comprise both not being at work (sick leave/absenteeism) as well as being at work but functioning sub-optimally (presenteeism). A recent literature review [36] found evidence to support an association between employees’ alcohol consumption and short-term as well as long-term sick leave, across socioeconomic status and gender. On a population level, Scandinavian time-series studies have linked increased alcohol consumption to increased sick leave. Based on alcohol sales in Sweden, it was estimated that a monthly increase of one decilitre pure alcohol per inhabitant was associated with 2–2.5 more long-term sick leave spells per 10,000 inhabitants [37], while an annual increase of 3.5 decilitres pure alcohol per inhabitant has been linked to an annual increase of 1.6 million sick leave days in the Swedish population [38]. A relationship between employees’ alcohol consumption and presenteeism (reduced on-the-job performance) has been demonstrated in several studies, e.g., in samples of American manufacturer employees [39], Finnish employees with multisite pain [40], Japanese community workers [41], and Norwegian employees in various occupations [42], implying that higher levels of alcohol consumption are associated with higher levels of work impairments. A Norwegian study [43] suggested that negative workplace consequences (e.g., safety and psychosocial issues) may occur even though the overall prevalence of alcohol-related abseenteeism and presenteeism may be quite low.

In addiction diseases, prevention is always of benefit. Alcohol prevention programmes targeting employees comprise a variety of intervention approaches on individual as well as an organisational level. According to Frone [21], they can be described as “interventions aimed at changing environmental, cultural, social, or personal factors in an effort (a) to keep individuals from abusing alcohol ( …) and (b) to avert adverse work outcomes” (p. 143), for instance in the form of workplace health promotion programmes or drug testing. Although evidence is somewhat mixed, certain intervention approaches (e.g., brief interventions consisting of one to four consultations) have demonstrated promising results [44–47]. Implementation of alcohol prevention programmes has, however, proved difficult [48], suggesting that providing health professionals with research evidence and/or clinical guidelines may not be sufficient. Rather, evidence must be combined with implementation strategies aimed at providing health care professionals with encouragement and skills necessary to change established routines [49].

Implementation of brief alcohol prevention programmes has mainly been studied in primary care settings. Barry et al. [48] found that lack of time was the most important barrier to implementation. In a review of qualitative evidence [50], it was concluded that successful implementation is dependent on adequate financial and managerial support combined with workload reduction and training opportunities for health care professionals. In a sample of nurses working with hospitalised patients, lack of alcohol-related knowledge and skills, concerns about negative patient reactions and logistic issues (e.g., lack of time) were found to be salient anticipated barriers to implementation of alcohol prevention programmes [51]. Similarly, Babor et al. [52] concluded that lack of time, staff turnover and competing priorities were associated with lower alcohol prevention activity.

Research related to OHS practice is limited, and research on alcohol prevention in the OHS is particularly sparse [1, 9, 53–55]. There is a need for further research on alcohol prevention in the OHS and on OHS professionals’ potential role in increased prevention of alcohol problems [1]. In order to develop strategies aimed at enabling implementation of alcohol prevention programmes in the OHS, it is pivotal to gain knowledge about which barrier domains should be targeted. Implementation barriers may originate from and reside within different domains or contexts, such as the OHS’ organisation itself (e.g., resources, time, workload, and competence/training), or factors external to the OHS’ organisation (e.g., employers’/clients’ interest in focusing on employees’ alcohol consumption, individual factors relating to OHS professionals’ or employers’/clients’ personal attitudes). Different barrier domains may require different implementation strategies and, moreover, different barrier domains may relate dissimilarly to working with different alcohol risk groups (e.g., primary, secondary and tertiary prevention activities). Hence, there is a need for studies investigating relationships between alcohol prevention activity and implementation barriers, i.e., for studies that explore associations beyond merely asking OHS professionals to rate which implementation barriers they perceive to be most salient. The present study adds to existing literature by providing updated knowledge on a rather under-researched topic, by generating knowledge on associations between implementation barriers and alcohol prevention activity, not merely on which and to what extent professionals perceive barriers, and by recognising that relationships between implementation barriers and prevention activity may vary according to alcohol risk level.

The primary aims of this study were to explore current practices of alcohol prevention targeting employees in occupational health settings, and examine whether and which perceived implementation barriers were associated with alcohol prevention activity. The secondary aim was to explore whether implementation barriers were differentially associated with primary, secondary and tertiary prevention activities.

## Methods

### Design and setting

The present study was designed as a cross-sectional survey as part of the Norwegian national WIRUS-project (Workplace Interventions preventing Risky Use of alcohol and Sick leave). Other results from the WIRUS-project are published elsewhere [24, 29, 42]. The study was conducted in 2018 among 357 health care professionals in 69 OHS units in Norway. OHS in Norway is regulated by the Working Environment Act [56] and OHS’ are accredited by the Norwegian Labour Inspection Authority, based on having at least three OHS professionals with expertise in the field of systematic health, safety and environmental (HSE) work (systematic activities undertaken in order to secure and improve the work environment), such as occupational hygiene and medicine, ergonomics and psychosocial work environment [3]. Systematic HSE work constitutes an interdisciplinary field, and the most frequent educational backgrounds among OHS professionals in Norway are nursing, medicine and physical therapy [57]. The proportion of employees in the Norwegian workforce who has access to OHS coverage is approximately 60%, which is somewhat higher than in the USA, but quite comparable to other European countries [2]. In Norway, Akan represents an organisation that plays a key role in handling issues related to alcohol, drugs, gaming and gambling among employees [58]. Exploration of the role of Akan is beyond the scope of this study.

### Data collection and sample

Contact information for accredited OHS’ was obtained from the Norwegian Labour Inspection Authority, and all 206 accredited OHS’ were invited to participate in the study. Ninety-three (45.2%) OHS’ responded to the invitation. Twenty-four of the 93 responding units declined to participate, and 12 of these units provided the following reasons for declining the invitation: Nine units did not have capacity to participate in research due to high workload, two units declined due to being involved in reorganisation processes, and one unit perceived the study as irrelevant to them. Sixty-nine units (74.2% of the responding OHS’) agreed to participate and sent lists of contact information for all health care professionals in their OHS. OHS’ from all geographical counties in Norway were represented in the study. Moreover, OHS’ providing services for companies in all work divisions (based on Eurostat’s classification of economic activities [59]) were represented. Electronic questionnaires were distributed to 601 OHS professionals. A total of 357 (59.4%) responded, while 295 (49.1%) responded on all relevant items (20.0% males; 80.0% females), and thus constituted the study sample. Respondents’ mean age was 49.1 years (*SD* = 9.9 years) and, on average, they had 12.3 years of experience as OHS professionals (*SD* = 9.1 years). A wide range of professions participated. Nurses (38.6%), physical therapists (17.3%), and physicians (13.9%) were the most frequent professions. Study sample characteristics are presented in Table 1.

**Table 1:** Characteristics of the study sample (*N* = 295)

				Range	
Variable	*M*	*SD*	Median	Min	Max
Age (years)	49.1	9.9	49.0	25.0	75.0
OHS experience (years)	12.3	9.1	10.0	< 1.0	39.0
Variable	*n*		%		
Gender					
Male	59		20.0		
Female	236		80.0		
Professional background					
Occupational therapist	8		2.7		
Nutritionist	1		0.3		
Physical therapist	51		17.3		
Physician	41		13.9		
Psychologist	6		2.0		
Nurse	114		38.6		
Occupational hygienist	23		7.8		
Other^a^	51		17.3		

*M* mean, *SD* standard deviation; ^a^ e.g., medical secretaries, engineers, educationalists/teachers, economists and social scientists

### Measures

#### Alcohol prevention activity

Respondents were asked to rate, on a five-point Likert scale (1 = not at all; 2 = to a small extent; 3 = to some extent; 4 = to a large extent; 5 = to a very large extent), to what extent their OHS unit engages in alcohol prevention targeting employees, separately for three prevention levels (primary prevention, targeting low-risk drinkers; secondary prevention, targeting moderate to high-risk drinkers; tertiary prevention, targeting high to dependence likely-drinkers). A sum score for overall alcohol prevention activity was computed by combining the scores for activities on all three prevention levels (potential range = 1–15). Categorisations of risk levels were based on WHO guidelines [31] (see Fig. 1).

#### Perceived barriers to implementation of alcohol prevention programmes

On a visual analogue scale ranging from 1 (to a very small extent) to 11 (to a very large extent), respondents were asked to rate the extent to which they perceived the following seven factors as barriers to implementation of alcohol prevention programmes in the OHS: (i) “alcohol is a personal/private matter”; (ii) “companies are not interested in employees’ alcohol consumption”; (iii) “companies counteract programmes targeting their employees’ alcohol consumption”; (iv) “lack of knowledge on the importance of alcohol prevention among OHS professionals”; (v) “lack of knowledge on how to conduct alcohol prevention programmes among OHS professionals”; (vi) “lack of time and/or resources”; and (vii) “others than the OHS are responsible for treating/intervening against employees’ alcohol consumption”.

The implementation barrier items were developed as part of the WIRUS-project, based on findings from previous research studying implementation of alcohol-preventive efforts in primary care settings [48, 50–52], and on three qualitative interview panels where nine OHS professionals were openly asked about barriers and facilitators for working with alcohol prevention in occupational health settings. Qualitative interview data was thematically analysed, resulting in categories corresponding to the seven implementation barrier items.

The implementation barrier items were subjected to an exploratory factor analysis (maximum likelihood approach with oblique rotation), resulting in a simple two-factor solution. The first factor (*OHS competence/time/resources*) contained barriers concerning OHS’ competence and resources (items iv; v; vi). The second factor (*employer/employee barriers)* consisted of barriers concerning employers and employees (items i; ii; iii; vii). Factor structure and internal consistency for the implementation barrier items are presented in Additional file 1.

#### Covariates

Respondents’ perceptions of whether employees’ alcohol consumption may be characterised as a public health challenge (*challenge perception*) were measured with a five-point Likert scale ranging from 1 (no, not at all) to 5 (yes, to a very large extent). Respondents’ personal attitudes toward alcohol and work-related drinking (*drinking social norms*) were measured with the Drinking Norms Scale [60] (mean score of seven items; low score = restrictive attitudes, high score = liberal attitudes). *Frequency of alcohol cases* (how often the OHS professional typically works with alcohol-related cases) was measured on a seven-point Likert scale (1 = never; 2 = less than yearly; 3 = yearly; 4 = less than monthly; 5 = monthly; 6 = weekly; 7 = daily). To what extent respondents believed OHS’ should focus on alcohol prevention targeting employees (*attitudes towards increasing alcohol prevention activity*) was measured on a Likert scale (1 = considerably less than today; 2 = less than today; 3 = same as today; 4 = more than today; 5 = considerably more than today), with the addition of a neutral category of “unsure”. Respondents also reported their *age* (years), *gender* (male; female), *OHS experience* (years) and *professional background* (occupational therapist; nutritionist; physical therapist; physician; psychologist; nurse; occupational hygienist; other).

#### Analysis

Descriptive statistics were utilised to analyse OHS professionals’ perceptions of employee alcohol consumption as a public health challenge, how often they typically work with alcohol-related cases, perceived implementation barriers, and the OHS’ alcohol prevention activity. One-way analysis of variance (ANOVA) was applied to explore whether frequency of working with alcohol-related issues differed according to professional background. Differences between alcohol prevention activity on different prevention levels were tested by means of paired samples t-tests. Multivariate linear regression analyses were used to investigate whether and how OHS’ alcohol prevention activity was associated with perceived implementation barriers. In order to allow meaningful comparisons between independent (predictor) variables, results from regression analyses were expressed in terms of standardised coefficients (β). Statistical procedures were utilised based on sample size and exploration of whether specific tests’ assumptions were appropriately met (e.g., the normality of data were tested by inspection of histograms, standardised residual plots, normal and detrended normal q-q plots). All statistical analyses were performed with IBM SPSS version 24. Significant results were defined as *p* < .05.

#### Ethics

OHS’ and respondents were informed about the study’s aim, assured confidentiality and that participation was voluntary. Written informed consent was obtained from all respondents. The study was approved by the Norwegian Centre for Research Data (NSD; reference no. 58038). The study was carried out in accordance with relevant guidelines and regulations.

## Results

### Current practices of alcohol prevention

Eight out of ten (80.4%) OHS professionals agreed that employees’ alcohol consumption constitute a public health challenge (17.3% disagreed; 2.4% were unsure). However, seven out of ten (69.5%) reported that they typically worked with alcohol-related cases less than monthly (21.7% on a monthly basis; 8.8% on a weekly basis). Those who, to some extent, did work with alcohol cases did not differ from those who never worked with alcohol cases with regard to perception of OHS alcohol prevention activity and perception of implementation barriers (see Additional file 2: Table S2, 1). The reported frequency of working with alcohol-related cases differed significantly according to professional background (*F* [2, 287] = 12.4, *p* = <.001, η^2^ = 0.2). Alcohol-related issues were primarily handled by physicians (*M* = 4.4; *SD* = 1.1), psychologists (*M* = 4.3; *SD* = 1.4) and nurses (*M* = 4.0; *SD* = 1.4), with a mean case frequency corresponding to between “less than monthly” and “monthly”. Occupational therapists (*M* = 2.9; *SD* = 1.7), physical therapists (*M* = 2.7; *SD* = 1.5), and occupational hygienists (*M* = 1.9; *SD* = 1.1) were to a smaller extent involved in alcohol prevention, with a mean case frequency corresponding to between “less than yearly” and “yearly”.

Overall, alcohol prevention activity were quite limited within the OHS’ (only one out of ten OHS professionals worked with alcohol-related cases on a weekly basis). In their prevention activities, OHS’ were most focused on tertiary prevention (*M* = 3.3; *SD* = 0.8), followed by secondary prevention (*M* = 2.9; *SD* = 0.7) and primary prevention (*M* = 2.8; *SD* = 0.8). The difference between tertiary and primary activities was statistically significant, t (294) = 8.9, *p* = <.001. Similarly, the difference between tertiary and secondary activities was significant, t (294) = 10.0, *p* = <.001. The difference between primary and secondary activities was not significant, t (294) = − 1.4, *p* = .17. OHS’ alcohol prevention activity, according to prevention level and differences between levels, are presented in Table 2.

**Table 2:** Alcohol prevention activity according to prevention level, and matrix of differences between prevention levels (*N* = 295)

	Primary activities (*M* = 2.8; *SD* = 0.8)	Secondary activities (*M* = 2.9; *SD* = 0.7)
Primary activities (*M* = 2.8; *SD* = 0.8)	–	*M*_diff_ = 0.1^ns^ *p* = .173 t (294) = − 1.4
Secondary activities (*M* = 2.9; *SD* = 0.7)	*M*_diff_ = 0.1^ns^ *p* = .173 t (294) = − 1.4	–
Tertiary activities (*M* = 3.3; *SD* = 0.8)	*M*_diff_ = 0.5* *p* = <.001 t (294) = 8.9	*M*_diff_ = 0.5* *p* = <.001 t (294) = 10.0

Results from paired samples t-tests; *M* mean, *SD* standard deviation, *M*_diff_ mean difference; * Statistically significant difference (*p* < .05); ^ns^ Statistically non-significant difference (*p* > .05)

Almost seven out of ten (67.1%) OHS professionals agreed that OHS’ should focus more on alcohol prevention targeting employees (12.3% disagreed; 20.3% were unsure).

### Implementation barriers and associations with prevention activity

**Fig. 2:**
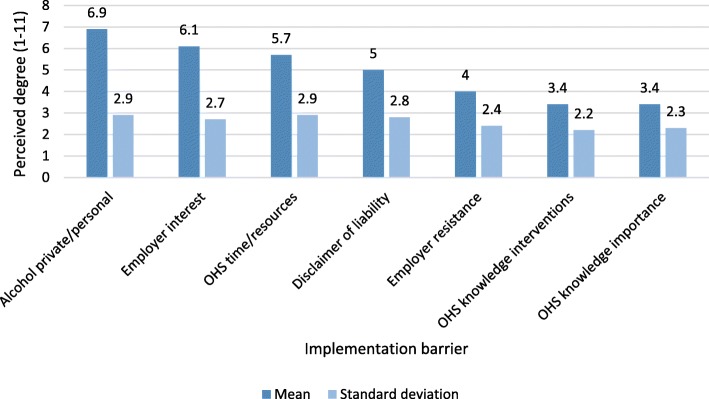
Perceived barriers to implementing alcohol-preventive efforts in occupational health services (*N* = 295). Means and standard deviations. Visual analogue scales ranging from 1 (barrier to a very small extent) to 11 (barrier to a very large extent)

When asked which barriers to alcohol prevention in the workplace were perceived as most salient, OHS professionals focused on alcohol being a personal/private matter (*M* = 6.9; *SD* = 2.9), and lack of employer interest in targeting their employees’ alcohol consumption (*M* = 6.1; *SD* = 2.7). An implementation barrier importance ranking is presented in Fig. 2.

Results from analyses of associations between perceived implementation barriers and alcohol prevention activity are presented in Table 3.

**Table 3:** Associations between perceived implementation barriers and alcohol prevention activity, overall and differentiated according to prevention level (*N* = 295)

Alcohol prevention activity				
Implementation barriers	All groups β (*p* value)	Primary β (*p* value)	Secondary β (*p* value)	Tertiary β (*p* value)
OHS competence, time, resources^a^	−0.22** (.001)	−0.20** (.002)	− 0.14* (.034)	−0.17** (.008)
Employer, employee^b^	−0.03^ns^ (.624)	−0.04^ns^ (.527)	− 0.03^ns^ (.651)	−0.01^ns^ (.945)

Results from multivariate hierarchical linear regression analyses; All models are adjusted for gender, age, professional background, OHS experience and drinking social norms; β = standardised coefficient; ^a^Barriers internal to the OHS’ organisation (items: “lack of knowledge on interventions”, “lack of knowledge on importance”, “lack of time/resources”); ^b^Barriers external to the OHS’ organisation (items: “lack of employer interest”, “employer resistance”, “alcohol is a private/personal matter”, “disclaimer of liability”); *p < .05; **p < .01; ^ns^Non-significant (*p* ≥ .05)

Barriers concerning OHS competence, time and resources demonstrated statistical significant associations with alcohol prevention activity, both overall (β = − 0.22; *p* = .001) and across all prevention levels. All associations were negative, implying that higher levels of perceived barriers were associated with lower reported prevention activity. With regard to specific prevention levels, OHS competence and resources were most strongly associated with primary prevention activities (β = − 0.20; *p* = .002), followed by tertiary (β = − 0.17; *p* = .008) and secondary prevention activities (β = − 0.14; *p* = .034). Reported employer/employee barriers were not significantly associated with alcohol prevention activity.

## Discussion

The primary aims of this study were to explore current practices of alcohol prevention targeting employees in occupational health settings, and examine whether and which perceived implementation barriers were associated with alcohol prevention activity. The majority of OHS professionals agreed that employees’ alcohol consumption constitute a public health challenge (eight out of ten), and that OHS’ should increase its prevention activity (seven out of ten). However, alcohol prevention activity was quite limited (seven out of ten worked with alcohol-related cases less than monthly, while only one out of ten did so on a weekly basis), and current activity was significantly more focused on tertiary prevention than on primary and secondary prevention. These findings are consistent with previous research that has emphasised that the OHS should be more actively involved in alcohol prevention [1, 5, 6, 22].

Detrimental health and work performance outcomes related to alcohol consumption are well documented [10–13, 36–42], and reducing harmful use of alcohol has been defined as a keystone in sustainable development [12]. Promotion of employees’ safety and health are emphasised in the aims of the OHS [1–3]. Hence, positive attitudes toward increased alcohol prevention in the OHS are not so surprising. Overall low prevention activity and favouring tertiary over primary and secondary prevention activities, may both be understood in terms of how the larger health care system is designed. The OHS do not operate in isolation from the health care system. Despite an increased awareness of benefits associated with preventive medicine and public health interventions, the health care system still tends to favour treatment (tertiary activities) over prevention (primary and secondary activities) [61]. According to Marvasti and Stafford [62], the health care system, designed in an era where handling infectious diseases was the major priority, is still today largerly characterised by an acute or reactive approach to health care. A system resting upon such a pathogenic paradigm [63] has been described as inexpedient in the current era where chronic and noncommunicable diseases (largely affected by lifestyle factors such as alcohol consumption) constitute the greatest threat to public health [62]. That OHS’ in the present study were most focused on employees already experiencing adverse health consequences (tertiary prevention) was also reflected in the finding that alcohol-related cases were primary handled by physicians, psychologists and nurses.

Descriptively, OHS professionals reported alcohol being a private/personal matter for employees as the most salient barrier against alcohol prevention activity, followed by lack of employer interest in targeting their employees’ alcohol consumption. Hence, when asked to identify and rank implementation barriers on a purely descriptive basis, our sample emphasised barriers related to employees and employers. However, analyses of associations between implementation barriers and alcohol prevention activity did display a quite different picture. Barriers concerning employers and employees (e.g., alcohol as a private/personal matter for employees, and lack of employer interest) were not significantly associated with alcohol prevention activity. In contrast, barriers internal to the OHS’ organisation (competence, time and resources) demonstrated significant associations with activity on all prevention levels, implying that lack of knowledge on the importance of working with alcohol and training in administering alcohol prevention programmes, as well as lack of time and resources, were associated with low alcohol prevention activity. This finding is in line with research studying barriers against implementation of alcohol prevention programmes in primary care settings [48–52], and implies that successful implementation strategies should involve not only an emphasis on individual OHS professionals, units, employees and employers. Facilitation of successful implementation of alcohol prevention programmes in the OHS may hinge on emphasising both inner (organisational level) and outer (system level) contextual factors [64, 65] in order to ensure adequate training, time and resources.

The present study does not contain data that can enlighten the observed discrepancy between the descriptive and analytical findings regarding implementation barrier perception. Overall, OHS professionals were in agreement on the importance on working with alcohol prevention. At the same time, they did express quite limited prevention activity. It is possible to conceive that an organisational-level self-serving bias may have played a role in explaining why the main barriers were attributed externally (to employees and employers) rather than to the OHS’ themselves. Self-protective attributional strategies is considered normal cross-cultural social-psychological phenomena [66, 67], and have also been identified within organisations [68]. The identified discrepancy does underscore the importance of studying implementation barriers beyond merely asking respondents to rate which barriers they perceive to be most salient.

The secondary aim of this study was to examine whether implementation barriers were differentially associated with primary, secondary and tertiary prevention activities. Results showed that implementation barriers were similarly associated with alcohol prevention activity on all three levels (i.e., that internal OHS barriers were related to prevention activity while external barriers were not). Hence, we found no fundamental reason to assume that different barriers apply when working on different prevention levels. Adequate training, resources and time stand out as important priorities in order to increase the implementation of alcohol prevention programmes in the OHS, regardless of whether they target individuals within the frames of primary, secondary or tertiary prevention.

### Methodological considerations

The present study has some limitations. Conducted within a cross-sectional design, exploration of causal relationships was not possible in this study. The aims were, however, related to investigating current practices and associations between variables. Thus, a cross-sectional design was deemed appropriate.

Results are based on data from 295 OHS professionals in 67 different OHS’. Of the 206 OHS’ contacted, 113 did not respond to the invitation and 24 declined to participate. In order to explore possible selection bias more thoroughly we have, on an organisational level, compared data from the included OHS’ with a representative sample of OHS’ included in a Norwegian official evaluation from 2016 [57] (see Additional file 3: Table S3, 1). With the exception of an overrepresentation of physical therapists in our sample (17.3 versus 9.4%, *p* < .05), distributions of professional background were not significantly different. OHS’ size (number of employees) and number of employers served by the OHS’ were not significantly different, with the exception of a few more OHS’ in our sample serving between 2 and 49 companies (28.8 versus 13.0%, *p* < .05). OHS’ from all geographical counties in Norway, providing services for companies across work divisions, were represented in this study. On an individual level, 59.4% (*n* = 357) responded to the questionnaire, while 49.1% (*n* = 295) were included in the study as a result of responding on all relevant items. Of those 62 not responding on all relevant items, 57 did respond to the sociodemographic items. With the exception of these 57 non-responders having somewhat shorter OHS experience than the study sample (median 7.0 versus 10.0 years, *p* < .05), the non-responders did not differ significantly with regard to age, gender or professional background (see Additional file 3: Table S3, 2). The gender distribution was quite skewed in this study (males: 20.0%; females 80:0%) but does correspond with the actual gender distribution among employees in health and social services in Norway (males: 19.0%; females: 81%) [69]. Moreover, male and female OHS professionals in our sample did not differ with regard to perception of OHS alcohol prevention activity and implementation barriers (see Additional file 2: Table S2, 2). Although we do not have reasons to believe that our sample was substantially non-representative, selection bias may constitute a possible limitation for this study. Hence, generalisations should be made with some caution.

The sample size was deemed satisfactory for analysing associations between variables as a result of well exceeding a recommended ratio of 15 participants per predictor variable [70], as well as exceeding the required size according to the formula *N* > 50 + (8 × *number of predictors*) [71].

In order to avoid losing statistical power, some OHS professionals who reported not to work with alcohol-related cases (*n* = 42) were included in the analyses, which may be perceived as a potential limitation. However, a series of additional tests did reveal that those professionals who did work with alcohol cases did not differ significantly from those who never worked with alcohol cases with regard to perception of OHS alcohol-preventive efforts and perception of implementation barriers (see Additional file 2: Table S2, 1).

Alcohol prevention activity and implementation barriers were measured by means of items developed particularly for the present study, which may be a limitation insofar that the instruments have yet to be validated. However, responses on all items were provided in the format of well-established response scales (Likert scales and Visual Analogue Scales). Moreover, the implementation barrier items were based on previous research as well as results from three qualitative focus group interviews with OHS professionals.

### Implications

The present study implies that current practices of primary and secondary alcohol prevention activities in the OHS are quite limited. This seems particularly true for primary prevention activities. Our identification of significant associations between implementation barriers and alcohol prevention activity across all prevention levels, and the fact that barriers were most strongly associated with primary prevention activities, imply that (i) an increase in overall alcohol prevention activity, and (ii) a shift from mainly focusing on tertiary activities to an increased emphasis on general health promotion and early intervention (primary and secondary activities), may both be dependent on adequate training of OHS professionals as well as allocation of time and resources. Our findings suggest that strategies aimed at enabling implementation of alcohol prevention programmes in the OHS should place an emphasis on targeting barriers relating to the OHS organisation itself, and should take both organisational-level and system-level factors into consideration.

## Conclusions

Alcohol consumption is associated with detrimental health and work performance outcomes, and occupational health settings may be particularly serviceable for alcohol prevention programmes targeting employees. However, this study found that the OHS infrequently engage in primary and secondary alcohol prevention activities. Factors internal to the OHS emerged as barriers against primary, secondary and tertiarty prevention activity . By ensuring adequate training, time and resources in the OHS, one may release an abeyant asset for preventing alcohol problems among employees, and thus contribute to remedy a major public health issue.

The relationship between implementation barriers and alcohol prevention activity in the OHS should be studied more thoroughly, preferably by means of longitudinal designs that enable exploration of causal mechanisms, and with studies investigating implementation processes in OHS related to specific alcohol prevention programmes (such as face-to-face interventions versus digital/web-based interventions). Moreover, future research would also benefit from exploring facilitating factors as well as implementation barriers.

## Additional files

Additional file 1: Factor structure and internal consistency for the implementation barrier items. (XML 7 kb) (PDF 287 kb)

Additional file 2: Mann-Whitney U tests for possible differences between professionals who worked with alcohol cases and those who did not, and between male and female OHS professionals. (PDF 22 kb)

Additional file 3: Study selection analyses. (PDF 304 kb)

## Data Availability

Data from the WIRUS OHS study are available from the project owner (University of Stavanger, Faculty of Health Sciences, Department of Public Health, Research group Societal Participation in School and Work) by principal investigator and project manager Randi Wågø Aas on reasonable request.

## Abbreviations

ANOVA: Analysis of variance
b: unstandardised regression coefficient
HSE: systematic health, safety and environmental work
*M*: mean
*N*: sample size
NSD: Norwegian centre for research data
OHS: Occupational health services
*p*: probability value
*SD*: Standard deviation
WHO: World Health Organization
WIRUS: Workplace Interventions preventing Risky Use of alcohol and Sick leave
β: standardised regression coefficient
