# Supplementary material for: Current practices and perceived implementation barriers for working with alcohol prevention in occupational health services: the WIRUS OHS study
Source: Subst Abuse Treat Prev Policy. 2019 Jun 26;14:30. doi: 10.1186/s13011-019-0217-2 (PMC6595559; doi:10.1186/s13011-019-0217-2)
Supplement: Supplementary file 1 — Factor structure and internal consistency for the implementation barrier items. (XML 7 kb) (PDF 287 kb) [file 13011_2019_217_MOESM1_ESM.pdf]

Additional file 1: Factor structure and internal consistency for the implementation barrier items

Table A1

Factor structure and internal consistency for the implementation barrier items

	Pattern matrix		Structure matrix		
Item	F_1	F_2	F_1	F_2	Communality
(v) knowledge interventions	**1.03**	-0.08	**0.99**	0.38	0.99
(iv) knowledge importance	**0.90**	-0.04	**0.89**	0.37	0.79
(vi) time/resources	**0.48**	0.07	**0.51**	0.28	0.27
(ii) employer interest	-0.11	**0.98**	0.33	**0.93**	0.88
(iii) employer resistance	0.16	**0.54**	0.40	**0.61**	0.39
(i) alcohol private/personal	-0.04	**0.51**	0.19	**0.50**	0.25
(vii) disclaimer of liability	0.22	**0.25**	0.33	**0.35**	0.16

	F_1	F_2	Both
Eigenvalue λ (% explained variance)	3.05 (43.50)	1.31 (18.71)	(62.22)
Cronbach's α	0.80	0.68	0.77
Mean inter-item correlation	0.60	0.35	0.33

Factor structure generated with exploratory maximum likelihood extraction with oblique rotation; Kaiser-Meyer-Olkin measure of sampling adequacy (KMO) = 0.69; Bartlett's test of sphericity p <.001
