# Supplementary material for: Current practices and perceived implementation barriers for working with alcohol prevention in occupational health services: the WIRUS OHS study
Source: Subst Abuse Treat Prev Policy. 2019 Jun 26;14:30. doi: 10.1186/s13011-019-0217-2 (PMC6595559; doi:10.1186/s13011-019-0217-2)
Supplement: Supplementary file 2 — Mann-Whitney U tests for possible differences between professionals who worked with alcohol cases and those who did not, and between male and female OHS professionals. (PDF 22 kb) [file 13011_2019_217_MOESM2_ESM.pdf]

Additional file 2: Mann-Whitney U tests for possible differences between professionals who worked with alcohol cases and those who did not, and between male and female OHS professionals

Table A2,1

Mann-Whitney U tests for possible differences between professionals who worked with alcohol cases and those who did not

Variable	Group*	Mean rank	U (z)	*p*
Alcohol-preventive efforts (all groups)	Alcohol cases<br>No alcohol cases	150.55<br>132.65	4668.50<br>(-1.29)	$.20^{ns}$
Alcohol-preventive efforts (low-risk drinkers)	Alcohol cases<br>No alcohol cases	151.43<br>127.43	4444.50<br>(-1.84)	$.07^{ns}$
Alcohol-preventive efforts (at-risk drinkers)	Alcohol cases<br>No alcohol cases	149.38<br>139.68	4963.50<br>(-0.80)	$.43^{ns}$
Alcohol-preventive efforts (probl./heavy drinkers)	Alcohol cases<br>No alcohol cases	148.64<br>144.15	5151.50<br>(-0.35)	$.73^{ns}$
Impl. barriers (OHS competence/resources)	Alcohol cases<br>No alcohol cases	149.83<br>136.95	4849.00<br>(-0.91)	$.36^{ns}$
Impl. barriers (employer/employees)	Alcohol cases<br>No alcohol cases	149.38<br>139.69	4964.00<br>(-0.68)	$.50^{ns}$

* Alcohol cases: n = 253, No alcohol cases: n = 42; ns = non-significant

Table A2,2

Mann-Whitney U tests for possible differences between male and female OHS professionals

Variable	Group*	Mean rank	U (z)	*p*
Prevention activity (all groups)	Males<br>Females	147.38<br>148.15	6925.500<br>(-0.06)	.95^{ns}
Prevention activity (low-risk drinkers)	Males<br>Females	135.84<br>151.04	6244.50<br>(-1.33)	.18^{ns}
Prevention activity (at-risk drinkers)	Males<br>Females	149.84<br>147.54	6853.50<br>(-0.22)	.83^{ns}
Prevention activity (probl./heavy drinkers)	Males<br>Females	153.13<br>146.72	6659.50<br>(-0.57)	.57^{ns}
Impl. barriers (OHS competence/resources)	Males<br>Females	136.30<br>150.93	6271.50<br>(-1.18)	.24^{ns}
Impl. barriers (employer/employees)	Males<br>Females	135.76<br>151.06	6240.00<br>(-1.23)	.22^{ns}

* Males: n = 59; Females: n = 236; ns = non-significant
