# Supplementary material for: Current practices and perceived implementation barriers for working with alcohol prevention in occupational health services: the WIRUS OHS study
Source: Subst Abuse Treat Prev Policy. 2019 Jun 26;14:30. doi: 10.1186/s13011-019-0217-2 (PMC6595559; doi:10.1186/s13011-019-0217-2)
Supplement: Supplementary file 3 — Study selection analyses. (PDF 304 kb) [file 13011_2019_217_MOESM3_ESM.pdf]

Additional file 3: Study selection analyses

Table A3,1

Characteristics of sample and occupational health services included in the study, compared with distributions reported in an official evualtion in Norway (Mandal et al., 2016[a])

**OHS professionals' background**	Study sample (N=295), % (n)	Mandal et al. (2016) (N=766), % (n)	Difference (*p* value)
Nurse	38.6 (114)	42.0 (322)	.314 ns[b]
Physical therapist	17.3 (51)	9.4 (72)	<.001*[b]
Physician	13.9 (41)	11.1 (85)	.206 ns[b]
Occupational hygienist	7.8 (23)	7.7 (59)	.959 ns[b]
Occupational therapist	2.7 (8)	3.8 (29)	.393 ns[b]
Psychologist	2.0 (6)	2.1 (16)	.955 ns[b]
Nutritionist	0.3 (1)	1.0 (8)	.458 ns[c]
**Number of employees in OHS**	Study sample OHS (N=56), % (n)	Mandal et al. (2016) OHS (N=163), % (n)	Difference (*p* value)
0-4	12.5 (7)	12.9 (21)	.941 ns[b]
5-9	50.0 (28)	46.0 (75)	.606 ns[b]
10-19	23.2 (13)	31.3 (51)	.252 ns[b]
20-49	12.5 (7)	6.1 (10)	.148 ns[c]
50-99	1.8 (1)	2.5 (4)	1.000 ns[c]
**Number of employers served by OHS**	Study sample OHS (N=59), % (n)	Mandal et al. (2016) OHS (N=169), % (n)	Difference (*p* value)
1	25.4 (15)	23.1 (39)	.715 ns[b]
2-49	28.8 (17)	13.0 (22)	.006*[b]
50-99	11.9 (7)	8.3 (14)	.413 ns[b]
100-199	10.2 (6)	20.7 (35)	.070 ns[b]
200-299	8.5 (5)	13.0 (22)	.352 ns[b]
300-399	3.4 (2)	8.9 (15)	.250 ns[c]
400-499	5.1 (3)	5.3 (9)	1.000 ns[c]
≥500	6.8 (4)	7.7 (13)	1.000 ns[c]

ns=non-significant; *significant ($p < .05$); [a]Mandal R, Dyrstad K, Melby L, Midtgård T. Evaluering av bedriftshelsetjenesten i Norge [Evaluation of the occupational health services in Norway]. Oslo, Norway: Sintef; 2016; [b]Difference tested with chi square test of independence; [c]Difference tested with Fisher's exact test

Table A3,2

Characteristics of study sample (N=295), compared to non-responders (N=57) in the survey

	Study sample	Non-responders[a]	Difference (p value)
**Age (years)**			.079 ns[b]
Median	49.0	47.0	
Range	25.0-75-0	28.0-65.0	
**OHS experience (years)**			.018*[b]
Median	10.0	7.0	
Range	0.0-39.0	0.0-30.0	
**Gender**			.856 ns[c]
Male, % (n)	20.0 (59)	21.1 (12)	
Female, % (n)	80.0 (236)	78.9 (45)	
**Professional background**			.074 ns[c]
Occupational therapist, % (n)	2.7 (8)	5.4 (3)	
Nutritionist, % (n)	0.3 (1)	0.0 (0)	
Physical therapist, % (n)	17.3 (51)	16.1 (9)	
Physician, % (n)	13.9 (41)	7.1 (4)	
Psychologist, % (n)	2.0 (6)	3.6 (2)	
Social worker, % (n)	0.0 (0)	3.6 (2)	
Nurse, % (n)	38.6 (114)	35.7 (20)	
Occupational hygienist, % (n)	7.8 (23)	8.9 (5)	
Other[d], % (n)	17.3 (51)	19.6 (11)	

ns=non-significant; *significant (p <.05); [a]OHS professionals who only responded to the sociodemographic items in the survey; [b]Difference tested with Mann-Whitney U test; [c]Difference tested with chi square test of independence; [d]E.g., medical secretaries, engineers, educationalists/teachers, economists and social scientists
